# Biophysical and Structural Characterization of Antibody–Drug Conjugates

**DOI:** 10.3390/cancers18060917

**Published:** 2026-03-12

**Authors:** Isabel P. Mariano, Abhinav Nath

**Affiliations:** Department of Medicinal Chemistry, School of Pharmacy, University of Washington, Seattle, WA 98195, USA; imariano@uw.edu

**Keywords:** drug development, therapeutic proteins, monoclonal antibodies, biologics, mass spectrometry, calorimetry, fluorescence, light scattering

## Abstract

Antibody–drug conjugates (ADCs) comprise a monoclonal antibody covalently bound to a cytotoxic payload by a linker. ADCs minimize off-target effects on healthy tissues, leveraging the specificity of monoclonal antibodies to deliver cytotoxic drugs to the intended tumor site. ADCs can be prone to poor behavior, including aggregation and misfolding, leading to poor efficacy, impaired pharmacokinetics, and immunogenicity. It is advantageous to understand the developability and potential liabilities of a protein candidate prior to costly in vivo studies or clinical trials. This review summarizes biophysical and structural techniques used to characterize ADCs and introduces emerging techniques aimed at accurately assessing the developability of protein candidates. Stability is commonly assayed using techniques like differential scanning calorimetry (DSC), differential scanning fluorimetry (DSF), or spectroscopic probes such as circular dichroism and intrinsic fluorescence. Drug-to-antibody ratio (DAR) is a critical parameter that can be measured using absorbance spectroscopy or chromatographic analysis. Aggregation and self-association can be probed using scattering techniques such as dynamic light scattering (DLS), static light scattering (SLS), and size exclusion chromatography–multi-angle light scattering (SEC-MALS), as well as more specialized approaches such as fluorescence correlation spectroscopy (FCS) and analytical ultracentrifugation (AUC). Mass spectrometry (MS) provides extremely valuable insight into stability, covalent modifications, and, through approaches like hydrogen–deuterium exchange (HDX-MS), structural dynamics of ADCs. Looking forward, the use of biophysical assays in ex vivo matrices and strategic use of artificial intelligence/machine learning (AI/ML) approaches are likely to advance the efficient and rapid development of ADCs and other next-generation protein therapeutics.

## Simple Summary

Common cytotoxic agents used to treat cancers can be accompanied by harsh side effects due to their ability to kill cells and systemic circulation. Antibody–drug conjugates utilize the high target affinity of antibodies to bind and release their cytotoxic payload at the tumor site. However, in order to create an effective treatment, biophysical characterization of antibody–drug conjugates is essential. Antibodies are prone to various undesirable behaviors including aggregation due to misfolding or weak interactions, and poor pharmacokinetics and disposition. The linker and cytotoxic payload add additional potential for misbehavior such as premature linker cleavage. By implementing biophysical characterization early in drug development, the detection of poorly behaved antibody–drug conjugates before expensive clinical trials is possible.

## 1. Introduction

Cancer is a leading cause of death around the globe, with an estimated 20 million new cases of cancer in 2022 and accounting for about one in six deaths worldwide [1]. For decades, the main method of treatment for a diverse range of cancers has been cytotoxic agents. Common cytotoxic agents used for chemotherapy include alkylating agents, antimetabolites, topoisomerase inhibitors, and mitotic inhibitors [2]. However, most chemotherapies are associated with severe side effects due to off-target and off-site toxicities damaging healthy tissues [3]. To overcome these challenges, there has been a large interest in creating cancer therapeutics that can achieve high targeting specificity against cancer cells. Antibody–drug conjugates (ADCs) are designed to take advantage of the specificity of monoclonal antibodies to effectively deliver cytotoxic agents to cancer cells while limiting systemic exposure and toxicities.

The US Food and Drug Administration approved the first ADC, Mylotarg (gemtuzumab ozogamicin), in 2000 for the treatment of acute myeloid leukemia in adults. ADCs have since become a promising avenue for targeted therapy in cancer, with 16 ADCs approved by the US Food and Drug Administration (FDA) as of March 2025 [4]. Moreover, more than 400 ADCs are in development globally, with over 200 in different stages of clinical trials [5].

**Figure 1:**
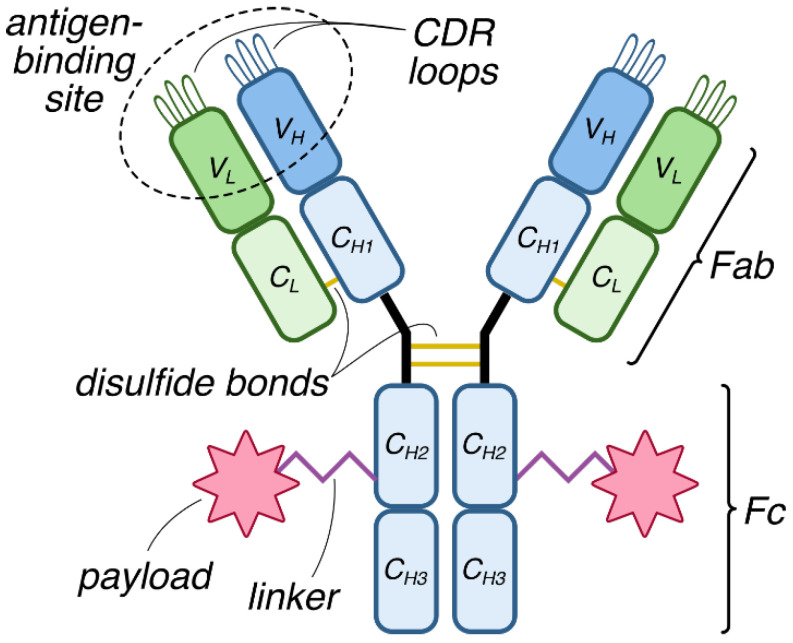
A schematic representation of an antibody–drug conjugate. The mAb portion of an ADC consists of two light chains (green) and two heavy chains (blue), covalently linked by disulfide bonds. The antigen binding site is located in the variable domains (V_L_ and V_H_), which each bear three loops that together comprise the complementarity-determining region (CDR). Cytotoxic payloads (purple stars) are attached to the mAb by linkers (purple lines), which may be cleavable or non-cleavable.

ADCs consist of a monoclonal antibody (mAb), a linker, and a cytotoxic payload, (Figure 1) making them very complex molecules prone to poor behaviors. Antibodies can be prone to aggregation due to intermolecular interactions between proteins. If these interacting proteins are involved in the immune system, anti-drug antibodies (ADAs) can arise due to the patient’s immune system producing antibodies against the therapeutic drug [6]. This response can lead to reduced effectiveness, increased clearance, and other adverse reactions [6]. The linker can cleave prematurely during circulation, leading to a loss of payload and potential off-target effects in healthy cells. Additionally, chemical modifications of both the payload and the mAb are possible, including oxidation of methionine, deamidation of asparagine and glutamine, and sulphation [7].

The vast majority of therapeutic antibodies, and all approved ADCs, are based on the immunoglobulin G (IgG) platform [8,9]. IgGs consist of two identical heavy chains (comprising a variable domain V_H_ and constant domains C_H1_, C_H2_ and C_H3_) and two identical light chains (comprising a variable domain V_L_ and a constant domain C_L_). The heavy and light chains are covalently bound by specific disulfide bonds that contribute to the remarkable stability of antibodies in vitro. ‘Hypervariable loops’ or complementarity-determining regions (CDRs) located in the V_H_ and V_L_ domains determine what antigenic target(s) will be engaged by any given antibody.

The misfolding behavior of antibodies and ADCs is a critical aspect of their pharmacokinetics and pharmacodynamics in vivo. As for many other proteins, the proper function of antibodies requires them to fold into a specific three-dimensional arrangement called the ‘native state’ [10]. ‘Misfolding’ is the failure of a protein to assume that correct native state. Misfolded proteins cannot carry out their proper functions and are at a greatly increased risk of forming protein aggregates. Individual domains of IgGs, when isolated from the larger antibody and studied in the laboratory, can unfold and refold reversibly [11]. However, unfolding behavior in the context of intact IgGs is more complex [12]. The constituent domains of an antibody interact with each other, and so one domain misfolding can destabilize another. Antibody unfolding is generally irreversible, and the resulting aggregates are immunogenic, eliciting the production of ADAs by the patient’s immune system [13]. Therefore, aggregation of therapeutic antibodies and ADCs in vivo or under formulation conditions (at concentrations typically well above 100 mg/mL) is a major liability [14].

During development, it is useful to understand the developability and potential liabilities of therapeutic protein candidates. Candidates that are prone to misfolding are at increased risk of displaying impaired pharmacokinetics, loss of efficacy, and immunogenicity leading to consequent toxicity [13,14]. There is great interest in identifying the biophysical characteristics that predispose mAb and ADC candidates to such undesirable behavior. As detailed below, a suite of techniques are available to interrogate different aspects of candidate structure and stability in vitro [15]. However, little is understood about complex matrices that therapeutic proteins are exposed to in vivo impact these parameters.

It is particularly important to understand these in vivo perturbations because mAbs, and any other therapeutics that bear an Fc fragment, circulate for extended periods of time, with half-lives typically in the 2–6 weeks range. Broadly speaking, mAbs and ADCs are cleared through endosomal–lysosomal degradation and, in many cases, by target-mediated drug disposition (TMDD) [16]. TMDD occurs when a significant fraction of the drug binds with high affinity to its target, and the resulting drug–target complex is then degraded. TMDD is a complex phenomenon that depends on the specific disposition pathways of the drug–target complex and the level of expression of the target, and frequently results in non-linear pharmacokinetics [17]. In contrast, internalization followed by degradation in endothelial cells is a more universal clearance pathway for therapeutic proteins. A key driver of slow disposition of therapeutic proteins is their rescue from endosomal–lysosomal degradation through binding to the neonatal Fc receptor (FcRn) [18,19]. FcRn is widely expressed in endothelial cells including in the liver and kidney, and binds human serum albumin (HSA) or Fc fragment-containing molecules in a pH-dependent manner. After HSA- or Fc-bearing therapeutic proteins are taken up by endocytosis, FcRn binds them strongly at the low pH (~5–6) of the maturing endosome. The FcRn–drug complex is then trafficked to recycling endosomes which fuse with the plasma membrane, where the higher extracellular pH (~7.4) induces FcRn to release the drug back into circulation. The affinities with which therapeutic proteins bind FcRn at both low and neutral pH are thus thought to be important determinants of pharmacokinetics, and a variety of Fc mutagenesis and engineering strategies are often used in attempts to engender longer half-lives in mAb and ADC candidates. In general, ADCs are cleared faster than mAbs, potentially due to decreased stability or increased non-specific or aberrant interactions in vivo caused by the presence of the linker and payload [20].

Biophysical and structural characterization is a critical aspect of understanding which ADC candidates are likely to be safe, effective, and well-behaved. The strategic use of biophysical characterization early in the developmental pipeline can help to flag potentially poorly behaved drug candidates prior to costly in vivo experiments and clinical trials. Such de-risking efforts will lead to more efficient development processes and, ultimately, safer and more effective ADC therapeutics for a variety of cancers. Here, we highlight key biophysical and structural parameters particularly relevant to ADC development.

## 2. Melting Temperature (T_m_)

**Figure 2:**
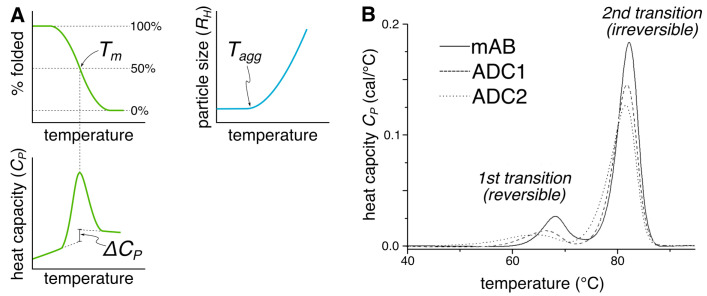
Measuring therapeutic protein stability. (**A**) Melting temperature T_m_ can be defined as the midpoint of the folding/unfolding transition, or the temperature at which the heat capacity peaks. Meanwhile, T_agg_ is the temperature at which a protein begins to aggregate, leading to an increase in hydrodynamic radius R_H_ and a decrease in diffusion coefficient D. (**B**) DSC measurements of mAb and ADC stability from Wakankar et al. [21]. The multiple peaks reflect the complex unfolding behavior of these proteins. The first transition is due to reversible partial unfolding limited to a few domains, while the second transition is due to irreversible complete unfolding. Conjugation with different payloads has a destabilizing effect in this case, decreasing the temperature at which partial unfolding begins.

T_m_ indicates the temperature at which a protein, or a domain of a protein, is 50% folded. A related but distinct parameter is the aggregation temperature (T_agg_), the temperature at which aggregation begins. Both parameters can both be used to indicate the thermal stability of a protein, and are useful in selecting stable proteins or formulations during development. As shown schematically in Figure 2, T_m_ can be measured by heating a protein and monitoring its unfolding via a spectroscopic signal (such as fluorescence or circular dichroism [CD]) or a calorimetric property such as the specific heat (the amount of energy necessary to raise the temperature of a sample by a defined amount).

Differential scanning calorimetry (DSC) is a widely used and powerful probe of protein thermal stability [22]. Energy is applied simultaneously to a sample cell containing the protein of interest and a reference cell containing only the solvent, such that the temperature of both cells increases at a constant, defined rate. The disparity in the input energy required to match the temperature of the sample cell to the reference cell is the excess heat absorbed by the protein in the sample, quantified in terms of the heat capacity (C_p_) [23]. Any phase transition such as the partial or complete unfolding of a mAb or ADC will require more energy to be applied to the sample cell. The resulting DSC scans, where the reference trace is subtracted from the sample, reveal how the excess heat changes as a function of temperature, and the transition midpoint is T_m_. The stability of the native conformation of a protein is determined by the Gibbs free energy (ΔG) of the system and the relationship between ΔH and changes in entropy (ΔS). The disruption of the forces stabilizing the native protein conformation, such as van der Waals, hydrophobic, and electrostatic interactions, leads to the unfolding of the protein. At high enough temperatures, where entropy is dominant, it overcomes the stabilizing forces, and unfolding of the protein occurs. DSC measures the change in enthalpy (ΔH) due to heat denaturation of a protein and can reveal factors that aid in the folding and stability of molecules [23]. The higher a protein’s T_m_, the more thermally stable the protein. DSC thus provides a much more detailed and mechanistically informative picture of any protein’s thermal stability. Kiyoshi et al. created an ADC using an engineered peptide for conjugation to the Fc and found enhanced antibody-dependent cellular cytotoxicity (ADCC) compared to the mAb [24]. Interestingly, when measuring the T_m_ for this ADC, the authors observed an increase in thermal stability following DSC analysis, with the conjugated Fc having a melting temperature of 88 °C compared to 70 °C and 82 °C for the CH_2_ and CH_3_ domains of the unmodified Fc [24]. The authors conclude that the Fc of the ADC has decreased molecular dynamics or entropy due to the conjugated peptide preventing Fc dynamics leading to the enhanced ADCC and increased thermal stability [24].

Differential scanning fluorimetry (DSF) also allows for the examination of protein unfolding and thermal stability by slowly heating a sample in a controlled environment and measuring changes in fluorescence. Extrinsic fluorescence utilizes dyes that are sensitive to their environment, such as dyes that are quenched in aqueous solutions, so that as a protein unfolds, the freely diffusing dye interacts with newly exposed hydrophobic surfaces and fluoresces. In intrinsic fluorescence, the source of fluorescence is the protein itself, typically from tryptophan residues. An example of a generally applicable approach is to measure intrinsic tryptophan fluorescence at 330 nm and 350 nm, with unfolding-induced changes in the microenvironment around the tryptophan residues typically causing a redshift in their fluorescence [25]. The T_m_ is calculated by measuring the ratio of fluorescence at 330 nm and 350 nm versus temperature [25]. Another approach is to fuse a fluorescent protein tag to a protein through a peptide linker, with the fluorescence signal from the tag reporting on its proximal environment [26]. However, the addition of a tag may perturb the unfolding behavior of the protein of interest. Karunarante et al. used both DSF and DSC to measure the thermal stability of the ADC of interest where the payload is conjugated to Cys215, near the hinge region [27]. Compared to the mAb, the ADC has a lower T_m_ for the CH_2_ domain as measured by DSC and for both the CH_2_ and Fab domains as measured by DSF, indicating a decrease in structural stability of the ADC upon conjugation to the payload [27].

Meanwhile, CD spectroscopy reports on the secondary structure content (α-helix and β-sheet) of a protein, and is relatively insensitive to amino acid composition. The CD signal is plotted against temperature, giving a sigmoidal curve that illustrates the fraction of unfolded protein with the inflection point of the curve, where 50% of the protein is unfolded, yielding the T_m_. Zhou et al. recently utilized CD to measure the thermal stability of their novel fully human single-domain antibody (UdAb) ADC where changes in CD signal at 216 nm were plotted against increasing temperature [28]. The measured T_m_ for their UdAb is 65 °C, indicating a thermally stable molecule, comparable to other UdAbs in their development pipeline [28]. CD is more commonly used to determine the secondary structure to an ADC following conjugation as seen in the study by Ebrahimi et al., who characterized the secondary and tertiary structure of an ADC across various formulations buffers [29]. A review by Mudhivarthi and Guo discusses CD as a biophysical technique to characterize ADCs [30].

## 3. Drug-to-Antibody Ratio (DAR)

DAR indicates the number of cytotoxic payload molecules conjugated to each monoclonal antibody and has a critical role in ADC design and efficacy. Typical DAR values can range from two, as in trastuzumab botidotin, to as high as eight, as in trastuzumab deruxtecan [31]. A larger DAR can appear more effective in vitro, but the plasma clearance of ADCs with higher DARs seems to increase compared to ADCs with lower DARs [20]. This observation could in part be due to the relationship between DAR and an ADC’s hydrophobicity [32]. A recent review by Matsuda and Mendelsohn describes the analytical approaches used for determining DAR in great detail [33].

### 3.1. Absorbance Spectroscopy

The simplest technique for determining the DAR of an ADC is to measure the absorbance of a sample at specific wavelengths close to the absorbance maxima (A_max_) of the mAb and the payload in the ultraviolet or visible spectrum [34]. The individual concentrations of the mAb and the payload can be calculated using the measured absorbance of the ADC and the extinction coefficients of the mAb and the payload at their respective A_max_ values (280 nm for mAbs). The molar ratio of the payload to antibody can then be calculated, giving the average DAR. This technique has been routinely applied to a wide variety of systems, including a conjugated vinca alkaloid, DAVLB (4-desactylvinblastine-3-carbohydrazide), where the A_max_ of the payload is only 10 nm different than that of the mAb [35]. Furthermore, McKertish et al. utilized UV-Vis analysis to determine the DAR of their dual-payload ADC [36]. However, in all studies using UV/VIS spectrometry to characterize DAR, the contribution of the payload and the linker to the measured absorbance of the ADC at 280 nm must be considered in the calculations of mAb concentration. Additionally, the result of these experiments is the average DAR in the sample, and the distribution of DAR is not known.

### 3.2. Chromatography and Mass Spectrometry

Chromatographic methods to characterize ADCs include hydrophobic interaction liquid chromatography (HIC) and reverse phase–high-performance liquid chromatography (RP-HPLC). Chromatographic separation can be used to quantify the average DAR and characterize the distribution of drug-linked molecules [33]. These techniques are particularly amenable to ADCs with distinct and limited conjugation sites. For example, the reduction in inter-chain disulfides creates free sulfhydryl groups, allowing for the conjugation of maleimide-containing linkers at specific sites [37]. This conjugation technique produces a mixture of ADCs with zero, two, four, six, or eight drugs per mAb [37]. During HIC analysis, ADCs are separated based on their hydrophobicity under native conditions, where the amount of conjugated payload will shift the hydrophobicity, and the DAR is calculated using the resulting chromatogram. Hamblett et al. utilize HIC to measure DAR and study its effects on the stability and efficacy of their anti-CD30 ADC in vitro and in vivo [38]. In reduced RP-HPLC, a reducing agent, such as DL-dithiothreitol (DTT), is used to break the interchain disulfide bonds between the heavy and light chains in antibodies. Separation by HPLC is facilitated by the difference in molecular weight and hydrophobicity between the light chain and the heavy chain. However, chromatography alone is limited by an inability to determine the molecular weight of each identified DAR population [33]. Intact RP-HPLC analysis for ADCs has been recently reported, including a recent development by Matsuda et al. where RP-HPLC was used to characterize cysteine-linked ADCs without sample pretreatment [39]. RP-HPLC analysis that maintains the intact antibody is especially advantageous because the use of a salt-free buffer system facilitates ionization efficiency allows it to be compatible with mass spectrometry analysis [39].

**Figure 3:**
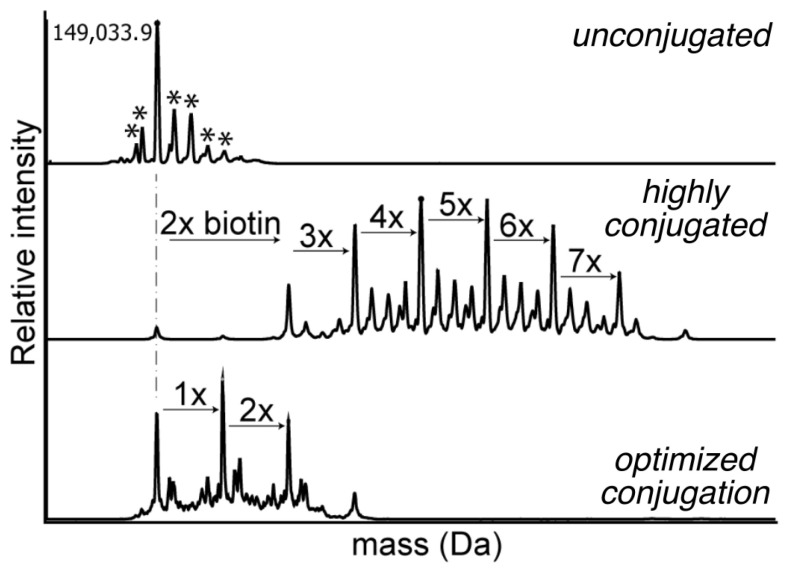
Characterization of mAb conjugation using intact mass spectrometry. Deconvoluted mass spectra are shown for sotrovimab before conjugation (**top**), or following extended (**middle**) or limited (**bottom**) conjugation with succinimidylester-biotin. The asterisks in the unconjugated spectrum indicate different glycoforms of the mAb. The dashed line indicates the mass of the predominant glycoform in the unconjugated sample. This demonstrates how intact MS can be used to characterize both endogenous covalent modifications and artificial conjugation of mAbs.

Native intact mass spectrometry (MS) is utilized to quantify the total antibody, free payload, and conjugated ADC. The deconvoluted mass spectrum of intact proteins gives information on the distribution of covalent modifications and posttranslational modifications (PTM), including the number of antibody drug conjugates and their relative abundance (Figure 3). The estimated payload amount and distribution of populations can be directly measured using intact MS data. A recent review by Beck et al. on advances in chromatography and mass spectrometry analysis for ADCs discusses the use of chromatography and native mass spectrometry to determine DAR as well as other applications of mass spectrometry in ADC analysis, including localization of conjugation sites and primary sequence characterization [15]. More specifically, Li et al. utilized native mass spectrometry to monitor the change in DAR and free drug concentrations following in vivo studies [40].

Identification of payload conjugation sites is completed using peptide mapping approaches where antibodies are digested into peptides by a protease, peptide digests are separated by liquid chromatography (LC), and MS analysis identifies peptides and conjugation sites. Chen et al. compared the two ADCs, ado-trastuzumab emtansine manufactured by two different companies, by their DAR, conjugation sites, and site occupancy [41]. DAR for the two ADCs was measured using intact MS with the candidate biosimilar ADC having a slightly lower DAR compared to Kadcyla. ADCs were treated with two different proteases, trypsin and Asp-N, the digests were separated by RPLC, and peptides were characterized by UV and MS analysis. Overall, the payload conjugation sites identified by the two different enzymatic digestions and between the biosimilar and the original ADCs were found to be consistent. To determine site occupancy, the authors compared the intensity of each conjugated tryptic peptide with an internal standard spiked in to normalize the MS signal intensity. Several sites in the biosimilar showed reduced or increased site occupancy compared to Kadcyla. Additionally, Beaumal et al. recently utilized MS analysis of trastuzumab deruxtecan to determine payload conjugation sites [42]. A recent review by Zhu et al. describes the current LC-MS-based methods for characterizing and quantifying ADCs, including DAR and payload-conjugation site analysis and quantification [43].

## 4. Target Affinity

The antibody component of the ADC should possess high target specificity, it should deliver the payload to the intended target, and it should have high binding affinity to the antigen. The dissociation constant (K_d_) is commonly used to describe the affinity between an antibody and an antigen, where the lower the K_d_, the higher the binding affinity. For antibodies, K_d_ is typically derived from association and dissociation rates as measured by various biophysical techniques, including surface plasma resonance (SPR), biolayer interferometry (BLI), and enzyme-linked immunosorbent assay (ELISA). A recent review by Lodge et al. describes the current techniques used to measure antibody affinity [44].

SPR, BLI, and ELISA are all high-throughput ligand-based techniques that rely on antibody or target immobilization to determine K_d_. SPR is an established technique utilizing label-free optical detection to measure the strength and rate of biomolecular interactions [45]. Onyido et al. describes a recent application of SPR to analyze antibody–antigen binding and cellular internalization in ADC development for ovarian cancer [46]. The authors successfully applied SPR to determine antibody–target pairs with ideal affinity and increased internalization that are overexpressed in ovarian cancer cells [46]. BLI is another label-free optical detection technique that can measure the association and disassociation rates of antibody–antigen binding. There are numerous applications of BLI to detect high affinity binding between ADCs and their respective antigens, including a recent identification of anti-GD2 ADC for treating solid tumors by Song et al. [47]. ELISA is also a common technique for measuring the K_d_ of an antibody–antigen pair, where one binding partner is immobilized while varying concentrations of the other are added. While all three techniques listed here can be employed to measure the K_d_ of an antibody–antigen pair for an ADC, each has advantages and disadvantages that must be considered when considering their utility. While ELISA measures the affinity constant thermodynamically, SPR and BLI utilize a kinetic measurement. Both SPR and BLI can measure binding in real-time; however, BLI is better suited for high-throughput screening and crude samples.

## 5. Self-Association and Aggregation

Self-association of antibodies can be responsible for high viscosity and aggregation, and it is especially important to characterize in the context of the high-concentration formulations necessitated by subcutaneous delivery. Self-association refers to all the weak, noncovalent forces resulting in net attractive interactions that can bring molecules together in a solution. Complications that can develop during the development of therapeutic antibodies, including ADCs, are high viscosity, liquid–liquid phase separation, and aggregation. While phase separation is reversible and aggregation is generally irreversible, both phenomena cause increased turbidity or opalescence [48]. High viscosity is a consequence of attractive intermolecular interactions, and may or may not be accompanied by phase separation or aggregation [49,50]. Self-association can lead to long-term stability issues, making it an important parameter to avoid in developability studies. Increased self-association can also lead to increased clearance or loss of efficacy of a therapeutic protein, due to recognition of the drug by the immune system. Protein aggregates may cause these adverse immunogenic effects resulting in a loss of therapeutic efficacy, making it essential to understand and predict if a therapeutic candidate is at risk for self-association [51,52,53,54,55].

### 5.1. Second Virial Coefficient (B_2_)

**Figure 4:**
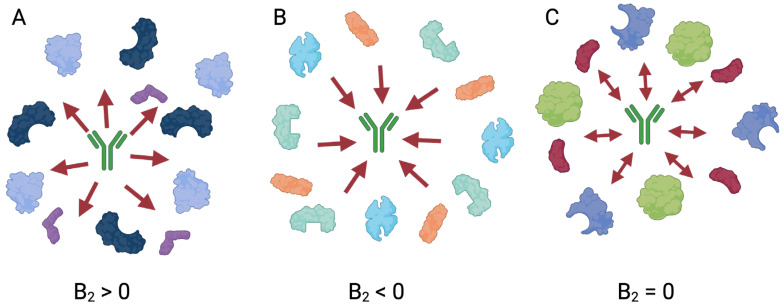
These illustrations depict potential net cosolute interactions represented by B_2_. Molecules of interest can participate in net repulsive interactions (indicated by outward-facing arrows) with a positive B_2_ (**A**) or net attractive interactions (indicated by inward-facing arrows) with a negative B_2_ (**B**). Attractive and repulsive interactions may balance each other out (indicated with double-headed arrows) with a B_2_ of zero (**C**).

The second osmotic virial coefficient, B_2_, measures weak, non-specific interactions between molecules in dilute solutions, with a positive value indicating repulsive interactions and a negative value indicating attractive interactions (Figure 4) [56]. It represents deviations from ideal behavior in solutions and quantifies the influence of pairwise interactions on the solution’s osmotic pressure [56]. B_2_ predicts protein crystallization, aggregation tendencies, and colloidal stability. The most common methods for measuring B_2_ involve detecting light scattering using either dynamic light scattering (DLS) or static light scattering (SLS).

In a DLS experiment, a monochromatic laser light illuminates macromolecules in a solution, and a detector measures the scattered light by the macromolecules [57]. By detecting and autocorrelating the fluctuations in scattered light due to the Brownian motion of macromolecules in a solution, DLS determines their translational diffusion coefficient (D_t_). In comparison, SLS involves measuring the intensity of light scattered over a range of angles from a sample. B_2_ affects the diffusion coefficient (measured by DLS) or the scattering intensity (measured by SLS) in a concentration-dependent manner, and so can be determined by measuring these parameters over a range of therapeutic protein concentrations. Ebrahimi et al. utilized DLS to study the conformational stability of the unconjugated mAb and the ADC, and concluded that the structural modifications present in the ADC drive increased protein–protein interactions [29]. However, by varying the buffer composition, the authors observed a reduction in turbidity and an increase in the diffusion interaction parameter k_D_ (a metric closely related to B_2_) reflective of a decrease in attractive protein–protein interactions [29]. Additionally, Johann et al. compared two ADCs with similar payloads that differed in their terminal group (neutral norephedrine moiety versus negative phenylalanine moiety with a terminal carboxylic acid group) to study how the properties of a payload can affect the colloidal stability and aggregation of ADCs [58]. The authors utilized SLS to measure B_2_ of the mAb and both ADCs, finding that both ADCs experienced significant increase in attractive interactions compared to the mAb [58]. The ADC with the negatively charged terminal group experienced a more significant decrease in the measured B_2_ compared to the mAb despite the payload being less hydrophobic [58]. Both studies highlight the importance of evaluating the effects of payload on ADC aggregation and colloidal stability, and how it can be used to guide the development of more stable ADC therapeutics.

Analytical ultracentrifugation (AUC) utilizes optical monitoring systems and ultracentrifugation to monitor a sample’s sedimentation profile in real time to measure its sedimentation coefficient [59]. A centrifugal field is applied, and the change in sample concentration is monitored versus the axis of rotation. The sedimentation coefficient is the ratio of a particle’s sedimentation velocity (SV) to the centrifugal acceleration. SV is measured by monitoring the radial concentration distribution of particles at time intervals throughout the course of an AUC experiment, and reports on the shape, molar mass, and size distribution of a sample. SV-AUC measurements over a range can be used to determine a sedimentation interaction parameter k_s_ that, with k_D_, can be used to rigorously determine B_2_ [60]. However, such SV-AUC measurements of k_s_ in complex, non-ideal mixtures require extremely careful analysis because the composition of such mixtures becomes non-homogeneous under centrifugation. This so-called Johnston-Ogston effect is a consequence of the fact that different constituents of a complex mixture will sediment at different rates, and so the analyte of interest will experience different types of interactions at different points in the sample [60]. SV-AUC also typically requires large amounts of sample, multi-hour experiments, and (if analyzing complex mixtures) fluorescent labeling of the protein of interest. These experimental challenges must be balanced against the unique insight that AUC can provide into therapeutic protein self-association. While by no means routine, SV-AUC has been used to characterize ADCs and related models [61,62,63].

### 5.2. Assays of Aggregation State

Size exclusion chromatography (SEC) separates proteins by their size, so it is used for estimating the size of macromolecules and to detect and quantify the presence of protein aggregates. A SEC column is packed with porous beads, so smaller macromolecules can penetrate the pores of the stationary phase, whereas larger molecules cannot enter the pores and elute off the column earlier. This technique requires that the sample not interact with the surface of the stationary phase, meaning that the elution times are based only on a molecule’s size or hydrodynamic radius and not electrostatic or chemical interactions. Detection of the sample as it elutes off the column is usually accomplished using UV absorbance, allowing for the detection and quantification of aggregation states in a protein sample. Johann et al. also utilized SEC to determine the extent of aggregation of the mAb and the two ADCs with differing terminal groups on their respective payloads following shaking and thermal stress [58]. Interestingly, the ADC with a negative terminal group in its payload displayed higher aggregation under both shaking and thermal stress, indicating that charge and hydrophobicity may be a reliable indicator of aggregation propensity. Additionally, Mills et al. determined the extent of aggregation at 2–8 and 40 °C over eight weeks of two ADCs in sixteen different formulations using SEC in addition to other high-throughput biophysical techniques [64]. The authors observed a reduction in aggregation following the addition of sugars and solubilizing agents at both pH 5.5 and pH 7 following storage at 40 °C, but the addition of salts and amino acids had differing effects, with them increasing aggregation at pH 5.5, while no destabilizing affect was observed at pH 7.

Light scattering is excellent at detecting even trace amounts of aggregates because scattering intensity rises sharply with particle size. SLS and DLS both provide additional valuable insight into self-association. SLS provides absolute measurements of molar mass and average molecular weight, whereas DLS measures the hydrodynamic radius and polydispersity of macromolecules and aggregates in solution. Both techniques provide information on colloidal stability and can detect aggregates in solution. SLS is excellent at detecting the moment aggregation begins, such as for measuring T_agg_, because it is a direct measurement of scattered light that will increase when average molecular weight increases. DLS is better suited for comparing differences in size between days or batches, and for measuring the change in size of particles in solution over long isothermal studies. Neither SLS nor DLS require fluorescent labeling of the sample, but are limited by their necessity for the sample of interest to be clear as well as the potential risk of multiple scattering. If a solution is colored and absorbs the laser’s wavelength, the measured intensity of the incident and scattered light will be misrepresented. SLS and DLS both rely on the principle that the scattered light has only been perturbed once by the sample particles. In opaque or highly concentrated solutions, photons can be scattered multiple times by particles in the sample before reaching the detector, making accurate measurements impossible. However, it is possible to overcome multiple scattering in SLS and DLS using cross-correlation [65,66]. While they lack the resolution of SEC, light scattering methods are faster and more amenable to high-throughput formats. Size exclusion chromatography–multi-angle light scattering (SEC-MALS), essentially SEC coupled to SLS detection, is an increasingly widely used approach that combines the analytical strengths of both SEC and light scattering.

## 6. Covalent Modifications

ADCs can undergo both physical and chemical modifications during manufacturing, processing, storage, and administration. Common covalent modifications include N-linked glycosylation, glycation, oxidation, deamidation, and isomerization. Such changes can result in alterations to mAb physical properties, including thermodynamic stability, hydrophobicity, secondary and tertiary structure, and charge, increasing the likelihood of aggregation and causing them to be susceptible to further chemical modifications. These modifications can also lead to immunogenicity and affect serum half-life. Chemical degradation of therapeutic proteins can occur throughout several phases in processing, manufacturing, storage, and administration.

Antibody glycosylation is a common PTM and is critical to antibody effector function and stability. MS techniques—often coupled to chromatographic separation—provide important insight into glycosylation state and glycan composition [67] (see example in Figure 3). Glycans are most commonly found on the Fc region of mAbs, with IgG1s having a single N-linked glycan at Asn^297^ in each of the CH_2_ domains [68]. However, during N-glycan synthesis, many sugar moieties can be added to create different glycoforms with particular glycoforms being necessary to achieve therapeutic efficacy [69]. Deglycosylation of IgG1s has been shown to lead to higher rates of aggregation, suggesting that the CH_2_ domain is involved in aggregation [70,71]. Furthermore, Fc N-glycans assist in Fc interactions with Fc receptors (FcR), influencing effector functions including ADCC and complement system activation. Removal of the Fc-glycan can eliminate complement activation and complement-dependent cytotoxicity (CDC), FcR binding, and ADCC [69,72,73,74]. The role of glycans in FcR binding and effector function has been extensively studied with a recent review by Majewska et al. summarizing its importance in therapeutic proteins [69]. Fc binding to the FcRn is thought to be independent of Fc glycosylation [75]. While the majority of glycosylation occurs in the Fc of mAbs, about 15–25% of mAbs also contain glycans within the variable region called Fab glycans [76]. A review by van de Bovenkamp et al. summarizes the potential role of Fab glycosylation during various physiological and pathological conditions, on IgG function, and immune modulation [76].

The quantification and composition of glycans is commonly achieved by coupling LC techniques, such as HILIC or RPLC, to a mass spectrometer. Glycosylation analysis can be divided into three approaches: released glycans, glycopeptides (bottom-up), and intact (top-down) and protein subunits (middle-up). Middle-down mass spectrometry includes the reduction of the ADC into subunits, which facilitates the recognition of mass shifts due to covalent modifications or loss of payload; however, it cannot localize modification sites. Bottom-up techniques enable site-specific characterization through peptide mapping addressing the limitations of middle-down mass spectrometry. A review by Duivelshof et al. details and compares the various methods applied to glycosylation analysis of biosimilars [67]. D’Atri et al. utilized middle-up HILIC MS analysis to study the glycan modifications of brentuximab vedotin, a cysteine-linked ADC [77]. Compared to RPLC, HILIC was effective in accurately separating the various glycoforms, allowing for simplified MS deconvolution and identification of less abundant glycoforms.

Glycosite-specific ADCs represent a newer platform where payloads are conjugated specifically to the glycan at Asn^297^. Recent studies on glycosite-specific ADCs have explored their stability in plasma and altered FcR binding. Hsu et al. created an anti-HER2 human and mouse IgG that utilized a bisecting glycan-bridge conjugation strategy and determined its linker stability in plasma [78]. The mouse ADC had about 65% of recovery of the intact ADC following a three-day incubation in rat plasma. However, decreased binding affinity was observed for the mouse and human ADC with FcγRI, FcγRIIa, and FcγRIIIa. An earlier paper by Zhu et al. found that anti-HER2 human antibody used to create a glycosite-specific ADC had similar binding affinity to FcγRI and FcγRIIIa as the unmodified IgG, while stability assays in human serum found no significant loss in activity after a 30-day incubation [79]. Taken together, it appears that site-specific conjugation of a drug moiety to the N-glycan in the Fc of IgGs can affect FcR binding and potentially inhibit effector function, but the effects depend on the chemical structure of the cytotoxic compound. Further research is needed to confirm this result with a broader range of ADCs and drug moieties.

Glycation is a frequent, nonenzymatic PTM of antibodies, where reducing sugars such as glucose and lactose, react with the ε-amino group of lysine residues and α-amino group of amino-terminal residues. Similar to glycosylation, glycation can affect the charge heterogenicity and aggregation formation of ADCs, leading to interbatch inconsistencies and functional activity affects. Xu et al. developed an improved MS method to monitor glycation of two therapeutic antibodies, and found a total of twenty-three potential glycation sites following forced glycation in several buffer conditions [80]. Additionally, Zhang and Du identified and quantified glycation of the cysteine-linked ADC trastuzumab deruxtecan, as well as a range of other PTMs including deamidation, oxidation, succinimide formation, and aspartic acid isomerization, using bottom-up MS analysis [81].

Oxidation and deamidation are the two most prevalent chemical degradation methods of mAbs. There are several routes through which oxidation can occur, including heavy metal ions, light, or the presence of oxygen [82]. Methionine and tryptophan are the amino acids most susceptible to oxidation, but other residues can be oxidized, including histidine, tyrosine, phenylalanine, and cysteine [83]. Bucheler et al. studied the impact of induced oxidation on model ADCs, finding that the specific conjugation technique used can influence the effects of oxidation [82]. Deamidation most commonly occurs at asparagine, but glutamine is also susceptible. The molecular mechanism of deamidation involves the formation of a succinimide intermediate that undergoes rapid hydrolysis, forming iso-aspartate or aspartate. Deamidation can affect the structure and function of mAbs by disrupting hydrogen bonds and/or instilling charge repulsion [84]. Cao et al. studied the effect of asparagine deamidation in the complementarity-determining region (CDR) of an ADC on its biological function [84]. The authors argue that the deamidation of an asparagine tryptophan motif within the CDR is dependent on the higher-order structure of the ADC, and deamidation can affect binding to the antigen and cytotoxicity. A review by Gupta et al. summarizes the effects of oxidation and deamidation on the stability, biological activity, and efficacy of mAbs, which can all be applied to ADCs [83].

The formation of a succinimide intermediate is not limited to the deamidation of asparagine and glutamine; aspartic acid and glutamic acid are also susceptible. The succinimide intermediate is short-lived in most cases, with deamidation of asparagine and glutamine or isomerization of aspartic acid being the end product. In some cases, the succinimide intermediate can accumulate, most likely due to formulation pH and conformation stability. VanAernum et al. discovered the accumulation of succinimide modifications in the CDR of the heavy and light chains of a therapeutic mAb using intact LC-MS [85]. The succinimide modification was found to decrease the mAbs’ potency, measured by binding ELISA, indicating the detrimental effects of succinimide accumulation on therapeutic antibodies’ efficacy. Isomerization of aspartic acid leads to the shortening of its side chain by one methylene group and elongation of the backbone by a methylene group. This change can affect the fold of the antibody near the isomerization site and cause a slight shift in its global charge. Huang et al. characterized the isomerization of aspartic acid in a pyrrolobenzodiazepine ADC using LC-MS based on the differing fragmentation patterns of aspartic acid and iso-aspartic acid following MS/MS fragmentation [86]. The authors observed a change in binding, suggesting that the isomerization of aspartic acid may lead to a change in tertiary structure indicating the importance of characterizing succinimide formation and the impacts it can have on ADC efficacy.

### Plasma Incubations

Thiol-maleimide linkers are one of the most common linker chemistries used in approved ADCs; however, they can react with free cysteine residues in human serum albumin (HSA), leading to albumin linker-payload adducts [87]. This albumin adduct can be long-lived, leading to off-target, off-tumor toxicities usually associated with adverse effects similar to cytotoxic payload. Other mechanisms of degradation to the linker include acidic degradation and cleavage by plasma and lysosomal proteases. In their review of the optimizing the safety of ADCs, Tarantino et al. discuss the potential toxicities associated with the cleavable and noncleavable linkers [88].

The incubation of ADCs in plasma or whole blood can provide insight into their stability and behavior in vivo. ADCs are incubated in human or another model species’ plasma/whole blood to observe how well the cytotoxic payload remains affixed to the antibody. These studies help to determine the stability of the linker during circulation and can predict if a drug is released prematurely. Following incubation in plasma or whole blood, liquid chromatography and/or mass spectrometry analysis are completed to quantify the amount of ADC versus free drug or unlabeled mAb. A recent study by Li et al. utilized plasma incubations followed by bottom-up and middle-down mass spectrometry approaches to inform ADC biotransformations [89]. Li et al. used the middle-down approach to evaluate the modifications to their original ADC following plasma incubations and determine the loss of conjugated payload indicating a discrepancy between the stability of the ADC versus the antibody [89]. To localize the modifications to their ADC, Li et al. captured the ADC following incubations in plasma and performed enzymatic digestion and peptide mapping analysis [89]. ELISA is another technique that can be utilized to measure loss of payload following plasma incubations. Pei et al. use ELISA to measure the concentration of their MMAE-conjugated ADC and total antibodies following incubation in sera [90].

Linker cleavage assays are necessary to understand the mechanism by which the payload is released from the ADC and inform on the controlled release of the payload to prevent off-target toxicities. Linkers can be grouped by the trigger for drug release: enzyme-liable drug release, acid-labile drug release, and redox-labile drug release. Mass spectrometry techniques, such as middle-down and bottom-up analysis, can be utilized to understand the extent of linker cleavage following exposure to their respective triggers. Dubowchik et al. analyzed the lysosomal release of doxorubicin linked to a monoclonal antibody via a dipeptide linker meaning it is susceptible to cleavage by proteases [91]. The authors incubated the ADC in various assay conditions designed to approximate the lysosomal environment and quantified the amount of free drug and ADC using RPLC. Yamaguchi et al. utilized LC-MS to analyze the cleavage of their bispecific ADC by observing the change in DAR following incubation with cathepsin B [92].

## 7. Conformational Perturbations

mAbs are dynamic molecules that can display altered secondary structure and flexibility upon covalent modification, ligand binding, and environmental changes. Hydrogen–deuterium exchange mass spectrometry (HDX-MS) is a high-resolution solution-based technique that has been used to probe such changes in protein conformation and dynamics in solution, protein–protein or protein–ligand interactions, and storage stability of protein-based therapeutics. HDX detects conformation changes and solvent exposure through the measurement of deuterium incorporation within the amide backbone, where amide hydrogens are exchanged for deuterons. Pan et al. utilized HDX-MS to investigate the structural integrity of site-specific ADCs [93]. Following a side-by-side comparison, Pan et al. found that the site-directed mutagenesis of Ser239 to cysteine did not impact the mAb’s HDX kinetics [93]. In the ADC, a small segment of the C_H_2 domain had a slightly increased HDX rate, indicating that conjugation of the drug only disrupts the local backbone amide hydrogen-bonding network involving Cys239 and site-specific conjugation of the drug does not impact the structural integrity of the mAb [93]. Furthermore, Cho et al. utilized solid-state HDX-MS to understand the structure and matrix interactions of an ADC with and without commonly used excipients under accelerated storage conditions [94]. HDX-MS can provide molecular insight into the effects of site-specific conjugation on the higher-order structure of ADCs, as well as identify favorable excipients for ideal storage stability.

## 8. Conclusions and Future Directions

To improve the therapeutic efficacy of ADCs, it is vital to understand their biophysical behavior during development, storage, and circulation within the patient. Current techniques discussed in this review are summarized in Table 1. To expand our knowledge of the behavior of ADCs and mAbs during circulation, ex vivo studies can be particularly informative while not as time- and resource-intensive as in vivo studies. Additionally, discrepancies between in vitro and in vivo results are often driven by the complexity of proteins, and ex vivo studies can help to bridge the gap. Due to the complex nature and behavior of proteins, which can be influenced by their environment, therapeutic proteins can behave differently in ideal, dilute buffer conditions typically associated with in vitro studies, compared to the highly complex and concentrated environments seen in vivo, such as serum and the endosomal lumen.

The direct study of antibody therapeutics’ biophysical behavior in serum or other biologically relevant solutions can improve our knowledge of the effect of crowded environments on pharmacokinetics and disposition. In recent work, our group has proposed the use of the apparent second virial coefficient, B_2,app_, of antibodies in serum to describe the changes in the balance of attractive and repulsive interactions using fluorescence correlation spectroscopy [56,95]. B_2,app_ is analogous to the self-association parameters described above (the traditional B_2_ and diffusion interaction parameter k_D_), with the distinction that B_2,app_ describes interactions between the therapeutic protein and other co-solutes that it encounters in vivo such as serum albumin, serum IgGs, complement factors, lipoprotein particles, etc. Negative values of B_2,app_ indicate net attractive interactions with co-solutes, which we hypothesize increases the risk of aberrant behavior in vivo. Just as the nature of drug payload, linker, and DAR can impact the stability of ADCs in vitro, they may also exacerbate the risk of problematic co-solute interactions in vivo. Such liabilities might not otherwise be detected until a candidate enters pre-clinical animal models or even clinical studies. We propose that B_2,app_ measurements on ex vivo samples could be used to de-risk therapeutic candidates earlier in development, to help explain inter-individual variability in therapeutic protein disposition, and as a method for early detection of individual patients’ ADA response or other immune reaction to a particular therapeutic.

The bioconjugation strategy used to connect the cytotoxic payload to the mAb continues to be an intriguing area of research, with emphasis on site specificity and linker stability. Non-natural amino acid incorporation is the insertion of a non-natural amino acid with a distinct functional group, which acts as a unique site for conjugation to the antibody [96]. While non-natural amino acid incorporation can guarantee site specificity and a consistent DAR, this technique is at risk of manufacturing issues related to low incorporation efficiency as well as potential immunogenicity [96]. THIOMAB technology, developed by Genentech, involves cysteine substitutions providing reactive thiol groups as a unique conjugation site to react with maleimide groups on the drug, allowing for an almost consistent DAR between batches due to the ability of maleimide to react with lysine [97]. Click chemistry can be used to manipulate and control the DAR of ADCs tightly. Click chemistry is characterized by the coupling of two unnatural functionalities that are orthogonal to other biological functional groups and whose products are stable in biological systems. Azide-alkyne cycloaddition is perhaps the best-known click chemistry reaction and has been used in ADC development [98,99,100].

Another exciting development is the widespread use of generative artificial intelligence and machine learning (AI/ML) methods to accelerate therapeutic protein development [101,102,103]. There has been substantial recent progress in AI/ML methods to design antibodies and de novo proteins for therapeutic applications [104,105]. However, computational optimization of therapeutic developability is still an open problem [106]. In silico simulations using molecular dynamics (MD) and related methods to predict properties such as therapeutic protein stability are computationally intensive [107]. AI/ML prediction of biophysical and pharmacokinetic properties of mAbs and other proteins shows promise, and can reveal unanticipated directions for more detailed experimental study [108]. For instance, we used regression on a small panel of antibodies to identify the change in heat capacity during unfolding, ΔC_p_, as a strong predictor of faster clearance in vivo [109]. However, the success of AI/ML approaches is dependent on the generation of large, high-quality data sets of relevant parameters. By combining these sophisticated inference approaches with new insights into how ADCs and other therapeutic proteins behave in vivo, it may be possible to make the development of these candidates faster and more efficient.

**Table 1:** Comparison of biophysical techniques used to measure T_m_, DAR, target affinity, aggregation, covalent modifications, and conformational changes.

Parameter	Technique	Advantages	Disadvantages
Melting temperature T_m_	DSC	Label-free Provides enthalpy changes and heat capacity	Low throughput Larger sample volume
	DSF	High throughout Small sample volume	Requires fluorescent dye
	CD	Label-free	Low throughput Sensitive to buffer
Drug-to-Antibody Ratio DAR	UV/VIS	Intact structure Relatively simple	Average DAR Requires clear solution
	Chromatography (HIC, RP-HPLC)	Intact or Digested Structure Distribution of DAR	Cannot identify peaks solely on retention time
	Intact MS	Intact or Digested Structure Distribution of DAR	Can be analytically challenging
Target Affinity	BLI	Real-time measurement Label-free Kinetic measurement High throughput	One binding partner immobilized Non-specific binding
	SPR	Real-time measurement Label-free Kinetic measurement	One binding partner immobilized Non-specific binding
	ELISA	High throughput Low cost	Plate-based immobilized Non-specific binding
Aggregation and Self-Association	DLS	Label-free Hydrodynamic radius, polydispersity, and B_2_	Requires clear solution and low concentrations
	SLS	Label-free Average molecular weight Well-suited for T_agg_	Requires clear solution and low concentrations
	AUC	Label-free Size distribution and B_2_	Requires large amounts of sample Multi-hour experiments
	SEC	Size distribution	Requires large amounts of sample
Covalent Modifications	LC-MS/MS	Nature, extent and location of PTMs	Can be analytically challenging
Plasma Stability	ELISA, LC-MS	Biologically relevant solution conditions	High background
Conformational Changes	HDX-MS	Solution-based structural technique High sensitivity	Low throughput Modest structural resolution

## Data Availability

No new data were created or analyzed in this study. Data sharing is not applicable to this article.

## Abbreviations

The following abbreviations are used in this manuscript:

ADA	Anti-drug antibody
ADC	Antibody–drug conjugate
ADCC	antibody-dependent cellular cytotoxicity
AI/ML	Artificial intelligence/machine learning
AUC	Analytical ultracentrifugation
CD	Circular dichroism
CDR	Complementarity-determining region
DAR	Drug-antibody ratio
DAVLB	4-desactylvinblastine-3-carbohydrazide
DLS	Dynamic light scattering
DSC	Differential scanning calorimetry
DSF	Differential scanning fluorimetry
HDX-MS	Hydrogen–deuterium exchange mass spectrometry
HILIC	Hydrophobic interaction liquid chromatography
HSA	Human serum albumin
LC	Liquid Chromatography
mAb	Monoclonal antibody
MALS	Multi-angle light scattering
MS	Mass spectrometry
PTM	Post-translation modification
SEC	Size-exclusion chromatography
SLS	Static light scattering
SV	Sedimentation velocity
UdAb	Fully human single-domain antibody
